# The role of the TGF-β1 signaling pathway in the process of amelogenesis

**DOI:** 10.3389/fphys.2025.1586769

**Published:** 2025-04-09

**Authors:** Xiaoxue Ma, Yunjing Ma, Zhiyong Lin, Mei Ji

**Affiliations:** ^1^ Department of Stomatology Shandong Provincial Hospital Affiliated to Shandong First Medical University, Shandong First Medical University, Jinan, Shandong, China; ^2^ Department of Stomatology Shandong Provincial Hospital Affiliated to Shandong First Medical University, Jinan, Shandong, China

**Keywords:** amelogenesis, TGF-β1, circadian clock, ameloblast, enamel formation, enamel development

## Abstract

Amelogenesis is a highly regulated process involving multiple signaling pathways, among which the transforming growth factor-β1 (TGF-β1) signaling pathway plays a pivotal role in enamel formation. This review firstly elucidates the critical functions of TGF-β1 in regulating ameloblast behavior and enamel development, encompassing ameloblast proliferation, differentiation, apoptosis, enamel matrix protein synthesis, and mineralization. Secondly, based on emerging evidence, we further discuss potential interactions between TGF-β signaling and circadian regulation in enamel formation, although this relationship requires further experimental validation. Finally, future research directions are proposed to further investigate the relationship between TGF-β1 and the circadian clock in the context of amelogenesis.

## 1 Introduction

Tooth enamel generated by ameloblasts is the most mineralized hard tissue in the human body (Xie et al., 2009; Yoshizaki et al., 2020; Zhong and Shibata, 2022). Enamel, derived from oral ectoderm, is the outermost layer of the tooth crown to protect the inner dentin and pulp from external damage and injury. Mature enamel consists of 95% minerals, 1%–2% organic materials, and 2%–4% water (by weight) (Deakins and Volker, 1941; Robinson et al., 1971; Schmitz et al., 2014). Since mature enamel is cell-free, once the enamel is damaged, it cannot be regenerated (Palmer et al., 2008). Its formation is mainly controlled by the interaction of epithelium and mesenchyme. The beginning of amelogenesis is marked by the differentiation of the inner enamel epithelium into ameloblasts at the late bell stage (Wang et al., 2020).

According to the morphology and function of ameloblasts, amelogenesis can be divided into different stages, including the prosecretory stage, the secretory stage, the transition stage, the maturation stage, and the protective stage (Reith, 1970). Among these, the secretory stage, the transition stage, and the maturation stage are the most important. During the secretory stage, ameloblasts are highly polarized with Tomes processes (Kallenbach, 1973; Lacruz et al., 2010). They can synthesize and secrete enamel matrix proteins (EMPs), such as amelogenin (AMELX), ameloblastin (AMBN), enamelin (ENAM), and various proteinases (Smith et al., 1996; Smith, 1998; Lacruz et al., 2010). Enamel matrix proteins are mainly processed by Matrix metalloproteinase 20 (MMP20) during this stage (Bartlett et al., 1998; Fukae et al., 1998; Caterina et al., 2002; Bartlett et al., 2011). After a short transition stage, the ameloblasts gradually become shorter, and the Tomes processes disappear (Kallenbach, 1974). The expression of enamel matrix proteins mentioned above is downregulated (Lacruz et al., 2012b). When the enamel reaches a certain thickness, it enters the maturation stage. In this stage, the morphology of ameloblasts is short and without Tomes processes. During this period, ameloblasts exhibit two different forms: ruffled ameloblasts and smooth ameloblasts (Smith, 1998). And the two forms can transform into each other every 8 h (Zheng et al., 2013). At this time, Kallikrein-related peptidase 4 (KLK4) processes and degrades enamel matrix proteins to promote enamel mineralization. Amelotin (AMTN) is also secreted to promote the final mineralization of the enamel matrix (Song et al., 2018).

When defects occur at any stage of amelogenesis, they can lead to the occurrence of amelogenesis imperfecta (AI) (Stephanopoulos et al., 2005). Amelogenesis imperfecta refers to a group of diseases characterized by hereditary developmental enamel defects. It may occur in isolation or may be associated with a syndrome. Amelogenesis imperfecta is mainly divided into hypoplastic and hypocalcified types (Sundell and Valentin, 1986). Once it occurs, the dentin and pulp are more susceptible to external stimuli and injuries, which can lead to corresponding diseases. Notably, mutations in genes associated with the TGF-β signaling pathway have been implicated in the pathogenesis of AI. For instance, loss of TGF-β1 in epithelium cells can result in AI in mice (Song et al., 2018). Additionally, targeted SMAD3 knockout in mice can lead to enamel hypomineralization (Yokozeki et al., 2003). Moreover, there is evidence that amelogenesis imperfecta has a negative impact on the oral health-related quality of life in affected individuals (Hashem et al., 2013).

**FIGURE 1:**
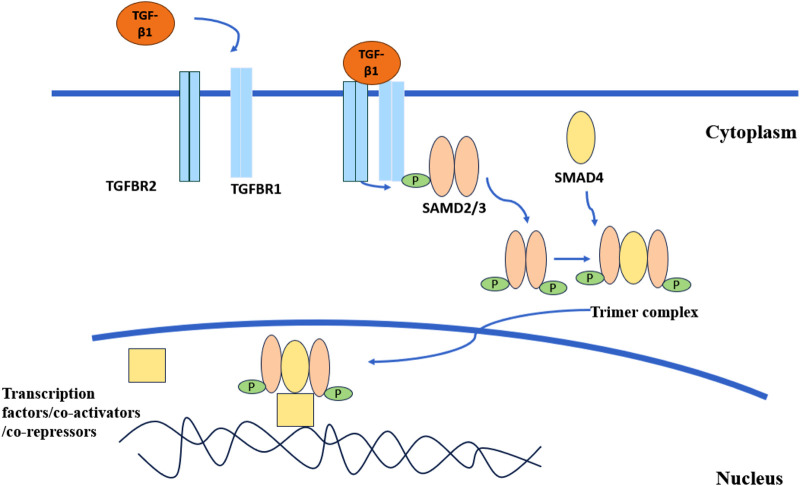
TGF-β classical signaling pathway. Activated TGF-β1 combines with TGFBR1 and TGFBR2 to form a complex. TGFBR2 phosphorylates and activates TGFBR1. The activated TGFBR1 binds to SMAD2 and SMAD3 and phosphorylates them. Phosphorylated SMAD2 and SMAD3 combine with SMAD4 to form a trimer complex. The complex is transported to the nucleus and jointly regulates the expression of specific genes with other transcription factors, co-activators, and co-inhibitors.

The TGF-β superfamily is a large class of extracellular growth factors divided into two subfamilies according to structural and biological criteria, namely the TGF-β/Nodal subfamily and the bone morphogenetic proteins (BMP) subfamily (Massagué and Sheppard, 2023). TGF-β is a multifunctional cytokine involved in the regulation of a variety of cellular processes, including cell proliferation, differentiation, apoptosis, and extracellular matrix formation (Moustakas and Heldin, 2005; Kubiczkova et al., 2012). There are three mammalian subtypes of TGF-β: TGF-β1, TGF-β2, and TGF-β3 (Kingsley, 1994). TGF-β1 is an important member of the TGF-β/Nodal subfamily (Kobayashi-Kinoshita et al., 2016). The TGF-β/SMAD signaling pathway is the classical TGF-β pathway (Figure 1). Many factors are able to activate TGF-β, such as reactive oxygen species, integrin, thrombospondin-1, pH, and so on (Lyons et al., 1988; Schultz-Cherry and Murphy-Ullrich, 1993; Barcellos-Hoff and Dix, 1996; Munger et al., 1998). Activated TGF-β1 binds to transforming growth factor beta receptor 1 (TGFBR1) and transforming growth factor beta receptor 2 (TGFBR2) to form a complex, in which TGFBR2 phosphorylates and activates the TGFBR1 kinase, and TGFBR1 binds and phosphorylates the transcription factors SMAD2 and SMAD3. Phosphorylated SMAD2 and SMAD3 bind to the universal SMAD4 to form a trimer complex, which is translocated to the nucleus and co-regulates the expression of specific genes with other transcription factors, coactivators, and co-repressors (Shi and Massagué, 2003). In addition, there are non-classical signaling pathways. In the non-classical pathways, TGF-β receptor complexes can transmit signals through other factors to participate in physiological processes such as cell differentiation, proliferation, apoptosis, and migration. Non-classical pathways include Notch signaling, MAP kinases, the AKT/PKB pathway, the GTP-binding protein pathway, the PTK pathway, NF-κB, the Wnt/β-catenin pathway, and so on (Kubiczkova et al., 2012). Dysregulation of the TGF-β signaling pathway is associated with many diseases, such as fibrotic diseases, cancer, connective tissue diseases, etc (Meng et al., 2016; van der Kraan, 2017; David and Massagué, 2018).

Previous studies have revealed that TGF-β subtypes are expressed at various stages of enamel formation (Table 1). In dental lamina and the bud stage, TGF-β1 was strongly expressed in the epithelial cells. In the cap stage, TGF-β1 was strongly expressed in the external enamel epithelium and stellate reticulum layer. In the bell stage, TGF-β1 was expressed in the external enamel epithelium, stellate reticulum layer, and internal enamel epithelium, and most of them were strongly positive, and TGF-β1 was also expressed in the subsequent ameloblasts (Sassá Benedete et al., 2008). In dental lamina, TGF-β2 was strongly expressed in some epithelial cells. In the bud stage, TGF-β2 was weakly expressed in the epithelial cells. In the cap stage, TGF-β2 was present in the stellate reticulum layer and inner enamel epithelial cells. In the bell stage, TGF-β2 was expressed in the external enamel organ, stellate reticulum layer, pre-ameloblasts, and ameloblasts (Sassá Benedete et al., 2008). There was no significant expression of TGF-β3 in dental lamina, the bud stage, and the cap stage. In the bell stage, TGF-β3 was weakly positive in the stellate reticulum and internal enamel epithelium cells, and positive in the odontoblasts of the dental papilla (Sassá Benedete et al., 2008). The expression of TGF-β1 at all stages of enamel formation provides evidence for the important regulatory role of TGF-β1 in amelogenesis.

**TABLE 1:** The expression of TGFβ subtypes in different stages of enamel formation.

Stages of enamel formation	Dental lamina	Bud stage	Cap stage	Bell stage
TGF-β1 expression	Strongly expressed in epithelial cells	Strongly expressed in epithelial cells	Strongly expressed in external enamel epithelium and stellate reticulum layer	Strongly expressed in external enamel epithelium, stellate reticulum layer, and internal enamel epithelium; ameloblasts
TGF-β2 expression	Strongly expressed in some epithelial cells	Weakly expressed in epithelial cells	Expressed in stellate reticulum layer and inner enamel epithelial cells	Expressed in external enamel organ, stellate reticulum layer, pre-ameloblasts, and ameloblasts
TGF-β3 expression	Not significantly expressed	Not significantly expressed	Not significantly expressed	Weakly expressed in the stellate reticulum and internal enamel epithelium cells; expressed in the odontoblasts of the dental papilla

The study finds that TGF-β1 plays an important role in multiple stages of amelogenesis. For example, TGF-β1 inhibited the proliferation of ameloblasts (Okubo et al., 2019), promoted the differentiation of ameloblasts (Coin et al., 1999), and induced the apoptosis of mature ameloblasts (Tsuchiya et al., 2009). In addition, TGF-β1 was shown to regulate the secretion of three major enamel matrix proteins and two proteases during enamel secretion (Okubo et al., 2019). Moreover, TGF-β1 also played an important role in promoting enamel mineralization (Yokozeki et al., 2003; Cho et al., 2013). Therefore, we aim to elucidate the regulatory roles of TGF-β1 on amelogenesis.

## 2 Regulation of ameloblasts by TGF-β1

### 2.1 TGF-β1 regulates the proliferation of ameloblasts

Cell proliferation is one of the important physiological functions of living cells and an important life feature of organisms. Cell proliferation is the basis of organism growth, development, reproduction, and heredity. TGF-β inhibits cell cycle progression by regulating the transcription of cell cycle regulators (Derynck and Zhang, 2003). TGF-β exhibits strong growth inhibition activity in a variety of cell types, and this inhibition is reversible after the removal of the ligand (Tucker et al., 1984; Ohta et al., 1987; Kimchi et al., 1988). Early evidence suggested that intravenous administration of TGF-β1 or TGF-β2 inhibited the proliferation of regenerated liver in rats (Russell et al., 1988). Experimental results showed that anti-TGF-β antibodies blocked TGF-β1-induced SMAD2 translocation and inhibited other effects of TGF-β1 in epithelial cells, such as P38 mitogen-activated protein (P38-MAP) kinase phosphorylation, cyclin kinase inhibitor expression, and DNA synthesis inhibition (Kawase et al., 2002). Thus, TGF-β1 might inhibit the proliferation of human oral epithelial cells through the SMAD2-mediated, p21WAF1/CIP1-dependent mechanism (Kawase et al., 2002). As described above, TGF-β1 can inhibit the proliferation of many cell types, such as regenerated liver cells, oral epithelial cells, etc. And the inhibitory effect of TGF-β1 is reversible.

Ameloblasts are terminally differentiated cells that do not normally proliferate once they have fully differentiated. During enamel formation, the primary role of ameloblasts is to secrete enamel matrix proteins and facilitate enamel mineralization, rather than to undergo cell division (Smith and Warshawsky, 1975). In the context of ameloblasts, TGF-β1 appears to exert a similar inhibitory effect on proliferation. Supporting evidence came from a previous study showing that TGF-β1 in dentin extract exerted an inhibitory effect on the proliferation of osteoprogenitor cells, suggesting that TGF-β1 may have the same effect on ameloblasts (Takata et al., 1998). A significant increase in odontogenic epithelial cell proliferation was detected after the knockdown of TGFBR2 (Chai et al., 1999). In the mature stage of enamel formation, the MTS assay (3-(4,5-dimethylthiazol-2-yl)-5-(3-carboxymethoxyphenyl)-2-(4-sulfophenyl)-2H-tetrazolium assay), a colorimetric method for assessing cell viability, was used to evaluate cell proliferation. The results showed that TGF-β1 and other subtypes inhibited the proliferation of mHAT9d cells, a dental epithelial stem cell line derived from the apical bud epithelium of a mouse incisor (Lee et al., 2024). This suggests that TGF-β1 plays a significant inhibitory role in ameloblast proliferation (Okubo et al., 2019). Furthermore, it was shown that ALC cells, an ameloblast-lineage cell line (Nakata et al., 2003), were very sensitive to TGF-β1-mediated proliferation inhibition *in vitro* (Tsuchiya et al., 2009). Taken together, TGF-β1 has a significant inhibitory effect on ameloblast proliferation, likely through mechanisms involving SMAD2 signaling and downstream cell cycle regulators. This not only reflects the role of TGF-β1, but also may be beneficial to the study of ameloblasts.

### 2.2 TGF-β1 regulates adhesion and differentiation of ameloblasts

During the secretory stage of enamel formation, ameloblasts change from cube-shaped to columnar, reorient polarity, and secrete enamel matrix proteins and peptidases, mainly including amelogenin, ameloblastin, enamelin, and MMP20 (Fukumoto et al., 2005; He et al., 2010). Many signaling pathways and molecules are involved in the regulation of ameloblast differentiation, such as Wnt/β-catenin, BMP4, and TGF-β1 (Balic and Thesleff, 2015; Miao et al., 2021). Some studies confirmed that TGF-β1 had a specific expression pattern during tooth development, and TGF-β1 was strongly expressed in the secretory ameloblasts, odontoblasts, stratum intermedium, stellate reticulum, outer enamel epithelium, and dental pulp, suggesting that TGF-β1 may play a crucial role in the differentiation of ameloblasts (Vaahtokari et al., 1991; Li and Pan, 2017).

The effect of TGF-β1 on the differentiation of different cells has been extensively studied. Prior studies confirmed the significant expression of TGF-β1 in hepatocellular carcinoma, breast cancer, and other cancers (Zarzynska, 2014; Zoheiry et al., 2015; Zhu et al., 2016). Moreover, TGF-β1 facilitated the proliferation, migration, and epithelial-mesenchymal transition of tumor cells (Wang et al., 2021). Under TGF-β1 stimulation, dental pulp cells were confirmed to be able to differentiate into odontoblasts and produce dentin in both *in vitro* and *in vivo* experiments (Li et al., 2011). The extracellular matrix of secretory enamel contained active TGF-β1, which induced the differentiation of human periodontal ligament cells (Nagano et al., 2006). In the study on organoids, it was demonstrated that the addition of TGF-β1 significantly enhanced the expression of enamel-related proteins in organoids and promoted the differentiation of organoids into functional ameloblasts (Hemeryck et al., 2022). Interestingly, the role of TGF-β signaling in ameloblast differentiation appears to be more complex than previously thought. A study demonstrated that conditional knockout of TGFBR2 in mice did not significantly affect ameloblast differentiation, despite the well-documented involvement of TGF-β signaling in epithelial cell differentiation and enamel formation (Oka et al., 2007). In conclusion, TGF-β1 can regulate the differentiation of tumor cells, dental pulp cells, periodontal ligament cells, etc., which prompts us to consider whether TGF-β1 regulates ameloblast differentiation.

The following studies have confirmed that TGF-β1 regulates the differentiation of ameloblasts. In transgenic mouse models, overexpression of TGF-β1 resulted in enamel defects, which were characterized by the shedding of ameloblasts from dentin and the formation of “cyst-like structures”. This suggests that TGF-β1 plays a crucial role in the differentiation and attachment of ameloblasts (Haruyama et al., 2006). Studies demonstrated that TGF-β1 induced ameloblast differentiation, particularly cell polarization, which was a key step in ameloblast differentiation and involves cell morphological changes and intracellular restructurings. It was verified that TGF-β1 in combination with heparin induced the differentiation of ameloblasts *in vitro* (Coin et al., 1999). When TGF-β1 was combined with LiCl (an activator of the Wnt/β-catenin signaling pathway) and epidermal growth factor (EGF), it enhanced the differentiation of ameloblasts. As a key regulator of various cellular processes, TGF-β1 might influence the differentiation of ameloblasts by regulating related signaling pathways such as the Wnt/β-catenin pathway (Miao et al., 2021).

In addition, studies verified that glycogen synthase kinase 3β (GSK3β) was implicated in the regulation of ameloblast differentiation through the Wnt and TGF-β signaling pathways (Yang et al., 2018). The inhibition of GSK3β resulted in the activation of the Wnt signaling pathway and the suppression of the TGF-β signaling pathway, thereby influencing ameloblast differentiation and enamel formation (Aurrekoetxea et al., 2016; Yang et al., 2018). Furthermore, the addition of TGF-β1 triggered the TGF-β pathway and subsequently significantly upregulated the expression of E-cadherin, AMBN, MMP20, and KLK4, which were markers of the adhesion and differentiation of ameloblasts (Yang et al., 2018). TGF-β1 induced the expression of AMBN in dental epithelial cells (Yang et al., 2018; Yamada et al., 2021). AMBN is an important regulatory factor in maintaining the differentiation of ameloblasts and is involved in the regulation of adhesion and proliferation of ameloblasts. In AMBN mutant mice, ameloblasts began to differentiate after leaving the matrix and dentin surface, but they quickly lost cell polarity, proliferated to form multilayer cells, and exhibited some characteristics of pre-ameloblasts (Fukumoto et al., 2005). In summary, TGF-β1 regulates ameloblast differentiation through related pathways (Wnt/β-catenin pathway and TGF-β signaling pathway). In addition, TGF-β1 alone or in combination with heparin, LiCl, and EGF can induce ameloblast differentiation and significantly upregulate the expression of markers of ameloblast differentiation. These findings reveal the complex regulatory network of TGF-β1 and its related molecular mechanisms in ameloblast differentiation.

### 2.3 TGF-β1 regulates apoptosis of ameloblasts

Apoptosis refers to programmed cell death, which plays a crucial role in development and tissue homeostasis. Studies have revealed that TGF-β is capable of inducing cell growth inhibition and apoptosis (Russell et al., 1988; Chai et al., 1999; Zimmerman and Padgett, 2000). TGF-β1 regulates the cell cycle and apoptosis by influencing the expression of cell cycle regulatory proteins (such as c-Myc, CDK4, and Cyclin D1) and apoptosis inhibitor proteins (such as p21Cip1) (Halder et al., 2005). In the process of enamel formation, these regulatory effects may have significant impacts on ameloblasts.

Many studies have proved that TGF-β1 exerts a regulatory effect on the apoptosis of ameloblasts. Approximately 25% of ameloblasts undergo apoptosis during the brief transition phase, and another approximately 25% are lost throughout the maturation period (Smith and Warshawsky, 1977). The expression of TGF-β1 was upregulated in mature enamel organs, and experiments confirmed that TGF-β1 treatment significantly enhanced the apoptosis of ameloblasts (Tsuchiya et al., 2009). In the maturation stage of enamel formation, the effect of TGF-β1 on apoptosis of mHAT9d cells was detected by immunohistochemistry, which indicated that TGF-β1 induced apoptosis of ameloblasts (Okubo et al., 2019). The mechanism underlying the apoptosis of ameloblasts might be as follows. Apoptosis of ameloblasts might be activated through two major molecular signaling pathways, intrinsic and extrinsic, both of which ultimately result in caspase activation and ultimate cell death (Abramyan et al., 2021). The upregulated expression of TGF-β1 in the mature ameloblasts might induce immediate early stress response genes through the TGF-β/activin signaling pathway in the maturation stage of amelogenesis, resulting in decreased expression of anti-apoptotic factor BCL2 and increased expression of pro-apoptotic factor BAX, thereby playing an important role in the apoptosis of ameloblasts (Tsuchiya et al., 2009). In general, it can be seen from the above that TGF-β1 can induce apoptosis of ameloblasts in the maturation stage.

In conclusion, during enamel development, the proliferation, differentiation, adhesion, apoptosis of ameloblasts, and the expression of related genes are all regulated by TGF-β1 to varying extents. It is speculated that the regulation of TGF-β1 may potentially be one of the crucial factors in guaranteeing the normal functionality of ameloblasts and the proper formation of enamel. However, it should be noted that the specific regulatory mechanism remains an area that requires further in-depth investigation and exploration. This is because the complexity and intricacy of the biological processes involved make it necessary to conduct more comprehensive and detailed studies to fully understand the precise workings and implications of this regulatory mechanism.

## 3 Regulation of enamel matrix synthesis by TGF-β1

The extracellular matrix plays an extremely crucial regulatory role in diverse aspects of cell signaling, function, characteristics, and morphology (Karamanos et al., 2021). It possesses the remarkable ability to exert an influence on cell proliferation, survival, migration, differentiation, and other cellular processes and functions (Lin and Bissell, 1993; Karamanos et al., 2021). During the secretory stage of enamel formation, ameloblasts synthesize and secrete enamel matrix proteins into the enamel region to constitute the enamel matrix. Among the numerous proteins within the enamel matrix, the most abundant structural proteins are amelogenin (AMELX), ameloblastin (AMBN), and enamelin (ENAM). These proteins play indispensable roles in the formation and development of enamel (Gibson et al., 2001; Lu et al., 2018; Wang et al., 2024). TGF-β1 is a key regulator that plays a vital role in the secretion of the enamel matrix. Its regulatory function is of great significance and has a profound effect on the entire process (Kobayashi-Kinoshita et al., 2016).

### 3.1 Regulation of amelogenin by TGF-β1

Amelogenin (AMELX) is required for normal enamel formation. By far, amelogenin is the most abundant component among all the enamel matrix proteins, accounting for approximately 90% (Termine et al., 1980), and it is also the protein that has been the subject of the most extensive research (Shaw et al., 2020). Two models of amelogenin guiding the growth of apatite minerals during the secretion stage are self-assembled nanospheres and nanoribbons (Paine and Snead, 1997; Bromley et al., 2011; Martinez-Avila et al., 2012). It is confirmed that amelogenin can promote calcium phosphate nucleation, promote the elongation of apatite crystals, stabilize calcium phosphate in solution, and inhibit its transformation into apatite *in vitro* (Moradian-Oldak and George, 2021; Pandya and Diekwisch, 2021). Amelogenin-deficient mice presented with chalky enamel that had a worn surface (Gibson et al., 2001). The thickness of this enamel was approximately one-sixth of the normal, and the hardness value was less than half of that of the wild type (Hu et al., 2016a). X-ray diffraction analysis showed that the main mineral in tooth enamel was octacalcium phosphate (OCP), rather than the commonly found hydroxyapatite (Hu et al., 2016a). The enamel failed to form a normal enamel band structure; instead, it had a fan-shaped mineralized structure, and the teeth appeared as calcified nodules (Gibson et al., 2001; Hu et al., 2016a). Mice lacking AMELX phosphorylation showed severe phenotypes, including enamel dysplasia, the absence of enamel columns, extensive ectopic calcification, an increased rate of conversion of amorphous calcium phosphate (ACP) to apatite crystals, and progressive cytopathology of ameloblasts (Gabe et al., 2024).

Some studies have confirmed the close relationship between TGF-β1 and amelogenin. During enamel formation, activated TGF-β1 binded to amelogenin cleavage fragments and maintained its activity. Subsequently, the complex binded to TGFBR1 to activate the TGF-β1 signaling pathway (Kobayashi-Kinoshita et al., 2016). Furthermore, research demonstrated the regulatory effect of TGF-β1 on AMELX; specifically, in TGF-β1 conditional knockout mice, there was an upregulation of AMELX expression during the maturation stage of ameloblasts (Song et al., 2018). However, the impact of TGF-β1 on amelogenin during the secretory stage of enamel formation remains elusive. Despite the existing evidence suggesting a potential regulatory role of TGF-β1 in amelogenin expression, the precise mechanisms by which TGF-β1 influences amelogenin secretion are not yet fully understood. Therefore, further studies are needed to explore this relationship in greater detail.

### 3.2 Regulation of ameloblastin by TGF-β1

Ameloblastin (AMBN), a multifunctional extracellular matrix protein in tooth enamel, plays a crucial role in various aspects of enamel development. It is involved in cell signaling and polarity, which are essential for the proper orientation and function of cells (Kegulian et al., 2024). AMBN also facilitates cell adhesion to the developing enamel matrix, providing a structural foundation for enamel formation (Fukumoto et al., 2004). Moreover, it contributes to the maintenance of prismatic enamel morphology, ensuring the structural integrity and aesthetic appearance of the tooth surface (Fukumoto et al., 2004; Kegulian et al., 2024). It regulates enamel matrix formation and mineralization by binding to hydroxyapatite crystals and maintaining the shape and size of these crystals, thus further influencing the overall development and quality of enamel (Lv et al., 2023b). As the second most significant enamel matrix protein, AMBN is associated with amelogenesis imperfecta when mutated (Poulter et al., 2014; Lu et al., 2018). And AMBN deletion leads to a complete lack of enamel formation (Liang et al., 2019).

The regulation of TGF-β1 on AMBN has been confirmed. Concrete evidence is described below. After treating LS8 cells, an immortalized ameloblast-like cell line (Chen et al., 1992), with exogenous TGF-β1, activation of the TGF-β1 pathway was found to increase the mRNA and protein abundance of AMBN and to activate the phosphorylation of SMAD2 and SMAD3. In addition, SIS3, an effective inhibitor of SMAD3, effectively blocked the upregulation of AMBN induced by TGF-β1, confirming the regulatory role of the TGF-β1/SMAD signaling pathway on AMBN (Lv et al., 2023b). Moreover, TGF-β1-induced AMBN expression was confirmed to be dependent on Connexin-43 (CX43) gap junction activity. When the gap junction was inhibited, TGF-β1-induced AMBN expression was blocked (Yamada et al., 2021). The underlying mechanism might involve CX43 facilitating calcium ion permeability between cells, leading to an increase in intracellular calcium ions. This increase promoted the conversion of Ras-GDP to Ras-GTP, which was upstream of ERK1/2 (Pérez-Sala and Rebollo, 1999), and subsequently enhanced TGF-β1-mediated phosphorylation of the ERK1/2 protein. Phosphorylation of ERK1/2 could facilitate the activation of runt-related transcription factor 2 (RUNX2). Once activated, phosphorylated RUNX2 binded to SMAD to initiate the transcription of target genes, including AMBN, which was essential for enamel development (Lai and Cheng, 2002; Franceschi and Xiao, 2003; Chen et al., 2012).

### 3.3 Regulation of enamelin by TGF-β1

Enamelin (ENAM) is the largest extracellular matrix protein produced by ameloblasts. It accounts for 1-5 percent of enamel matrix proteins and is recognized as the least abundant among the three major enamel matrix proteins (Hu et al., 2000; Hu et al., 2001). The cleavage products of ENAM play a pivotal role in the function of Tomes’ processes, the distal extensions of ameloblasts, which is crucial for the proper shaping, extension, and orientation of the mineral bands within the enamel (Simmer et al., 2021). Correspondingly, mutations in ENAM disrupted enamel formation, resulting in hypoplastic amelogenesis imperfecta. In severe cases, the enamel was almost invisible on the dentin surface (Yu et al., 2022; Wang et al., 2024). The study found that TGF-β1 conditional knockout mice led to the upregulation of ENAM expression in ameloblasts at the maturation stage (Song et al., 2018). However, further experimental evidence is necessary to confirm the precise regulatory mechanisms of TGF-β1 on ENAM.

To conclude, TGF-β1 is of great significance to the regulation of three major enamel matrix proteins, including AMELX, AMBN, and ENAM. TGF-β1 is closely related to AMELX and upregulates the expression of AMELX. In the case of AMBN, TGF-β1 has been found to increase the expression of AMBN. Enamelins’ role is also suggested to be regulated by TGF-β1, although the specific mechanisms require further elucidation. Since the synthesis and secretion of enamel matrix proteins are key to enamel formation, further study of the regulation of TGF-β1 on enamel matrix proteins will help to understand and explore the mechanism of enamel formation and treatment strategies for related enamel hypoplasia diseases.

## 4 Regulation of enamel matrix mineralization by TGF-β1

Many studies have shown that TGF-β1 plays an important role in enamel mineralization. Mice deficient in TGF-β1 exhibited severe enamel wear and a decrease in mineralized density, manifested by thinner enamel rods and the presence of enamel matrix protein residues (Song et al., 2018). Latent transforming growth factor beta-binding protein 3 (LTBP3) is required for the activation of TGF-β. The enamel defects observed in LTBP3 knockout mice underscored the significance of TGF-β1 in enamel mineralization (Morkmued et al., 2017). Furthermore, SMAD3 is known to be a central molecule in transforming growth factor-β (TGF-β) superfamily signaling, responsible for transmitting signals from cell surface receptors to the nucleus, thereby regulating gene expression. The hypomineralized enamel observed in SMAD3-deficient mouse models indicates that the TGF-β signaling pathway is critical for enamel formation and mineralization (Yokozeki et al., 2003). A study also demonstrated that the TGF-β1/SMAD3 signaling pathway was vital in alleviating fluorine-induced enamel hypomineralization (Bi et al., 2024).

In addition, exogenous TGF-β1 upregulated RUNX2 and tissue nonspecific alkaline phosphatase (ALP), thereby promoting enamel mineralization (Bi et al., 2024). Moreover, a study showed that TGF-β1 and RUNX2 had a synergistic effect on the regulation of enamel mineralization, and their co-loss led to more pronounced mineralization defects (Tian et al., 2022). The discovery of the important role of Forkhead Box O transcription factor FOXO1 in enamel biochemical mineralization further supports the hypothesis that the TGF-β1 signaling pathway regulates enamel matrix mineralization and maturation (Poché et al., 2012). FOXO1, as a transcription factor, might act synergistically with SMAD3 in response to TGF-β1 signaling and influence enamel maturation by regulating a common gene set. What’s more, intense staining of TGF-β1, connective tissue growth factor (CTGF), and phosphorylated-SMAD2/3 was observed in the reduced enamel epithelium, suggesting that the TGF-β1-SMAD2/3-CTGF signaling pathway may play a key role in enamel maturation (Li and Pan, 2018).

### 4.1 Regulation of peptidases by TGF-β1

Enamel mineralization is a process in which apatite crystals replace the enamel matrix. The degradation and absorption of the enamel matrix are primarily regulated by two peptidases, MMP20 and KLK4. MMP20, also known as enamelysin, is located on chromosome 11, while KLK4, a kallikrein enzyme, is situated on chromosome 19 and is crucial for the degradation of enamel proteins (Dong et al., 2023). It is suggested that MMP20 can activate KLK4, and KLK4 can inactivate MMP20 *in vitro* (Yamakoshi et al., 2013). During the secretory stage of enamel formation, MMP20 secreted by ameloblasts can promote the processing of enamel matrix protein to increase crystal thickness (Hu et al., 2002; Hu et al., 2016b). During the transition and maturation stages of enamel formation, secreted KLK4 actively degrades enamel matrix proteins to increase crystal width and thickness until the crystals come into contact with each other (Lu et al., 2008; Simmer et al., 2011). Studies of MMP20 and KLK4 functional deficiency mouse models found that MMP20 deficiency led to the inability of enamel crystals to thicken, and KLK4 deficiency led to enamel hypomineralization, pigmentation, and protein residues (Bartlett and Simmer, 2014; Hu et al., 2016b; Smith et al., 2017).

TGF-β1 can regulate enamel matrix mineralization by modulating the expression and activity of MMP20 and KLK4. TGF-β1 significantly promoted the expression of MMP20 in porcine dental pulp cells (Niwa et al., 2018). Consistently, TGF-β1 was shown to upregulate the expression of MMP20 in ameloblast-lineage cells (Gao et al., 2009). One study successfully established a mouse model of TGF-β knockout mice and found that MMP20 expression was significantly upregulated on postnatal day 7 (PN7) (Song et al., 2018). The regulation of MMP20 gene expression by TGF-β1 occurred through a mechanism that involved the transcription factor myocyte enhancer factor-2C (MEF2C) in ameloblast lineage cells (Gao et al., 2014).

In addition, studies have confirmed that TGF-β1 can promote the expression of KLK4. In TGF-β1 knockout mice, the expression of KLK4 was significantly downregulated, which affected the degradation of enamel matrix proteins and enamel mineralization (Song et al., 2018). In the study of the potential function of TGF-β isoforms in maturation-stage ameloblasts, TGF-β1 was found to significantly upregulate KLK4 mRNA levels (Okubo et al., 2019). In mice with a conditional knockout of the TGF receptor II, KLK4 RNA levels were significantly diminished, leading to reduced enamel mineralization, thinner enamel crystals, and increased wear, suggesting that enamel mineralization and maturation were regulated by TGF-β through the expression of KLK4 (Cho et al., 2013). Moreover, another study revealed that fluoride affected enamel protein content by inhibiting KLK4 through TGF-β1 *in vitro* and *in vivo* (Suzuki et al., 2014). The removal of the enamel matrix is a topic worthy of exploration. Failure to effectively remove the organic matrix of enamel can lead to low mechanical strength, wear, and even fracture of enamel, as seen in MMP20-null and KLK4-null mice (Caterina et al., 2002; Simmer et al., 2009; Yamakoshi et al., 2011).

### 4.2 Regulation of amelotin by TGF-β1

In addition to the two major peptidases mentioned above, the expression of Amelotin (AMTN) is also critical for enamel mineralization. AMTN is produced by ameloblasts and is expressed in the maturation stage of enamel formation. As a promoter of the mineralization of hydroxyapatite, AMTN can regulate the nucleation and growth of hydroxyapatite crystals (Abbarin et al., 2015; Danesi et al., 2021; Ikeda et al., 2022). AMTN deficiency led to delayed enamel mineralization, characterized by reduced mineralization of the inner enamel and structural anomalies of the outer enamel. These defects could compromise the enamel’s integrity and function, making it more susceptible to wear (Nakayama et al., 2015). The regulation of AMTN expression is a nuanced process that involves various signaling pathways. AMTN gene expression was regulated by TGF-β1 in gingival epithelial cells, suggesting a relationship between the two (Nakayama et al., 2019). Moreover, studies demonstrated that an increase in TGF-β1 activity significantly increased the synthesis of AMTN and played an important role in enamel mineralization (Okubo et al., 2019). In addition, in TGF-β1 conditional knockout mice, AMTN was significantly downregulated in enamel organs (Song et al., 2018). Collectively, the above scientific studies provide compelling evidence that TGF-β1 regulates the expression of AMTN.

### 4.3 Regulation of odontogenic ameloblast-associated protein by TGF-β1

Odontogenic ameloblast-associated protein (ODAM), initially identified as APIN, is expressed in ameloblasts during the maturation stage and in the junctional epithelium (JE) (Moffatt et al., 2008). ODAM and AMTN are sister genes, both of which originated from ENAM (Sire et al., 2007). Moreover, ODAM was found to play an important role in the enamel mineralization process by regulating MMP20 (Lee et al., 2010). Additionally, *in vitro* experiments confirmed that ODAM promoted the nucleation of hydroxyapatite (Ikeda et al., 2018). However, the research found that the formation and structure of enamel showed no significant changes in ODAM knockout mice (Wazen et al., 2015). More interestingly, a study on the functions of TGF-β subtypes revealed that TGF-β1 and TGF-β2 had no significant impact on the gene expression of ODAM, while TGF-β3 significantly downregulated the gene expression of ODAM (Okubo et al., 2019). Further exploration is still needed regarding the role of ODAM and the regulation of it by TGF-β1.

### 4.4 Regulation of endocytosis and ion transporters by TGF-β1

At the same time, in the maturation stage of enamel formation, TGF-β1 regulated the endocytosis of ameloblasts and promoted the uptake of enamel matrix proteins (such as amelogenin) by ameloblasts (Okubo et al., 2019). In addition, TGF-β1 influenced enamel mineralization by regulating RUNX2, which in turn affected the expression of the vesicle protein WD repeat-containing protein 72 (WDR72) (Liu et al., 2019). As an important regulator, WDR72 regulates the structure and assembly of microtubules in ameloblasts, which is crucial for the morphological changes of ameloblasts, endocytosis, and vesicle trafficking. All of these directly affect the pH regulation and mineralization of the enamel matrix (Liu et al., 2019).

Enamel formation is tightly regulated by extracellular pH, which is responsible for hydroxyapatite crystal growth and peptidase activity. A variety of ion transporters on ameloblast membranes are responsible for pH regulation (Ji et al., 2018). TGF-β1 has been shown to regulate pH by interfering with the expression of ion transporters to affect enamel mineralization. For example, sodium fluoride and sulfur dioxide derivatives downregulated the expression of the electrogenic sodium bicarbonate cotransporter NBCe1 (SLC4A4) by inhibiting TGF-β1, so that the acidified enamel microenvironment could not be fully neutralized by bicarbonate (Lv et al., 2023a). This interference could disrupt subsequent matrix synthesis, secretion, and mineralization (Lv et al., 2023a). TGF-β1 was also found to modulate the expression of voltage-gated chlorine channels ClC-5 and ClC-7 (Ji et al., 2021). These two channels are associated with tooth development and pH regulation and may be involved in the intracellular accumulation of fluoride (Ji et al., 2021). In addition, TGF-β1 was shown to downregulate the expression of cystic fibrosis transmembrane conductance regulator (CFTR) in colonic epithelial cells, thereby inhibiting chloride secretion (Howe et al., 2004). Similarly, TGF-β1 inhibited CFTR function in human bronchial epithelial cells (Snodgrass et al., 2013). Ameloblasts are typically epithelial-derived cells, so it is hypothesized that TGF-β1 may downregulate CFTR in ameloblasts. However, this hypothesis still needs to be confirmed by further experimental validation.

In addition to the above, the expression and function of TGF-β1 were regulated by the store-operated Ca2+ entry (SOCE) pathway (Said et al., 2020). In SOCE-deficient ameloblasts, TGF-β1 was significantly downregulated, and the phenotype of TGF-β1 receptor-specific knockout mice was similar to that of Stim1 conditional knockout mice (Said et al., 2020). Both phenotypes showed reduced enamel mineralization, diminished mineral content, and heightened enamel wear (Cho et al., 2013; Eckstein et al., 2017; Eckstein and Lacruz, 2018). It is suggested that the dysregulation of TGF-β1 may be partly involved in the amelogenesis imperfecta (AI) caused by the loss of Stim1. Furthermore, TGF-β1 acts as a circadian regulator. The disruption of SOCE alters the expression of TGF-β1, potentially impacting the circadian clock’s regulation of enamel thickness and mineralization, which are crucial for dental health (Said et al., 2020).

In conclusion, TGF-β1 signaling plays an indispensable and critical role in enamel matrix mineralization. The integrity of the TGF-β1 pathway is essential for proper enamel development, and any disruption in this pathway can lead to enamel hypomineralization or other enamel defects. TGF-β1 influences enamel mineralization through multiple mechanisms, including the regulation of Amelotin and matrix proteases such as MMP20 and KLK4. Additionally, TGF-β1 affects the endocytosis of ameloblasts, which is vital for the resorption of the organic matrix and the exposure of mineralized enamel to the oral environment. Furthermore, TGF-β1 regulates the expression of ion transporters, which are key for the movement of calcium and phosphate ions, the main constituents of enamel minerals.

## 5 Potential interplay between TGF-β1 and circadian clock

Circadian rhythms represent self-sustained endogenous oscillations occurring over a 24-h period. These biological rhythms play a critical role in modulating a wide range of physiological processes (Papagerakis et al., 2014). The circadian rhythms are controlled by the circadian clock. The circadian clock is divided into the central clock and the peripheral clock (Weaver, 1998; Hastings et al., 2003). At the molecular level, circadian rhythms are maintained through the differential expression of clock genes. The major clock genes in mammals include the positive regulatory genes such as circadian locomotor output cycles kaput (CLOCK), brain and muscle ARNT-like 1 (BMAL1), as well as the negative regulatory genes such as period (PER1, PER2) and cryptochrome (CRY1, CRY2) (Gekakis et al., 1998; Reppert and Weaver, 2002; Takahashi, 2017). The clock genes in cells interact with each other in a complex manner, forming self-regulating transcription-translation feedback loops (TTFLs) to control the rhythmic expression (Reppert and Weaver, 2002; Takahashi, 2017).

Numerous studies have investigated the critical role of the circadian clock in enamel formation. Previous research showed that the key circadian clock genes and the ameloblast stage-specific genes, including AMELX and KLK4, oscillated rhythmically in ameloblasts (Zheng et al., 2011; Lacruz et al., 2012a; Zheng et al., 2013; Huang et al., 2021). Additionally, enamel matrix proteins such as AMBN, AMTN, and ODAM also oscillated in a circadian pattern (Nirvani et al., 2017). Disruption of circadian rhythms in pregnant mice was found to delay enamel development in offspring, with the inner enamel epithelium failing to differentiate into high-columnar ameloblasts, suggesting that circadian rhythm is essential for ameloblast differentiation and enamel formation (Tao et al., 2016).

Further evidence highlights the mechanistic involvement of circadian genes in enamel secretion and mineralization. Overexpression of BMAL1 led to the upregulation of AMELX and KLK4 expression, implying that circadian rhythms may directly modulate these processes (Zheng et al., 2013). Conversely, PER2 knockdown not only reduced AMELX expression and ameloblast matrix secretion, but also altered the subcellular localization of E-cadherin, indicating that the circadian clock influences the adhesion and arrangement of ameloblasts (Huang et al., 2021). Moreover, the overexpression of the circadian clock gene Nuclear receptor subfamily 1, group D, member 1 (NR1D1) upregulated the expression of AMELX while down-regulating the expression of the MMP20 and KLK4 (Athanassiou-Papaefthymiou et al., 2011).

Interestingly, TGF-β1 has been associated with circadian rhythms in other tissues. Firstly, rhythmic expression patterns of TGF-β, SMAD3, and phosphorylated SMAD3 were observed (Beynon et al., 2009; Sato et al., 2012). Besides, in the cholestatic liver disease model of PER2 knockout, TGF-β1 was significantly increased (Chen et al., 2013). In pulmonary epithelial cells and normal lung fibroblasts, TGF-β1 can upregulate the expression of BMAL1. Additionally, the knockdown of BMAL1 significantly attenuated the canonical TGF-β1 signaling pathway (Dong et al., 2016). BMAL1 inhibited epithelial-mesenchymal transition through the TGF-β1/SMADs/Snail Family Transcriptional Repressor 1 (SNAIL1) axis, thereby enhancing the radio-sensitivity of nasopharyngeal carcinoma (Li et al., 2024). And under sleep deprivation stress condition, upregulation of BMAL1 and CLOCK inhibited TGF-β1 expression (Xing et al., 2024). Furthermore, BMAL1 positively regulated TGF-β1 in kidney fibrosis (Takaguri et al., 2025). Although the interaction mechanism between TGF-β1 and circadian rhythm in enamel formation remains unclear, the expression changes of TGF-β1 dependent on SOCE may affect enamel mineralization, which is known to be controlled by the circadian rhythm (Said et al., 2020). Future studies should test whether TGF-β1 interacts with core circadian clock genes during enamel formation.

## 6 Conclusion and future outlook

**FIGURE 2:**
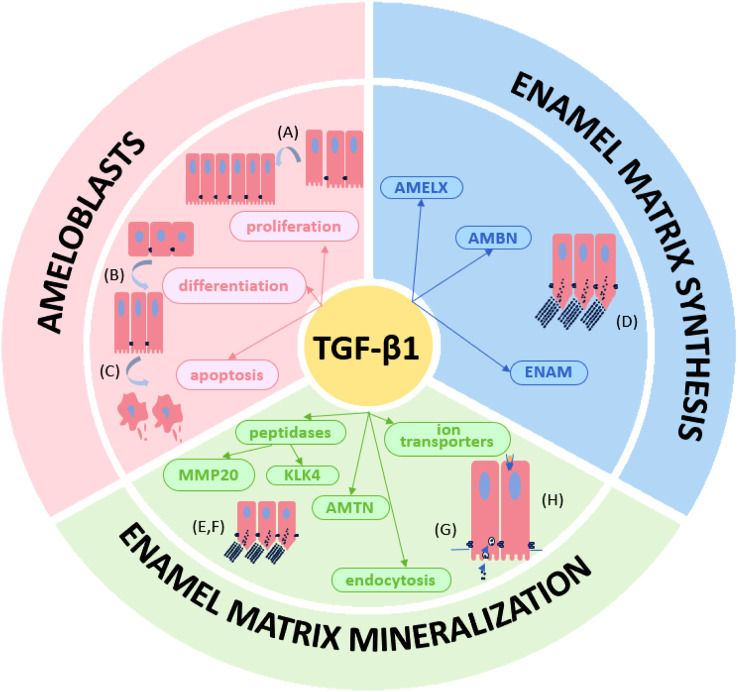
The TGF-β1 signaling pathway plays multiple roles in amelogenesis. The TGF-β1 regulates **(A)** proliferation, **(B)** differentiation and **(C)** apoptosis of ameloblasts. TGF-β1 also regulates in **(D)** enamel matrix (AMELX, AMBN and ENAM) synthesis and **(E–H)** mineralization (MMP20, KLK4, AMTN, endocytosis of ameloblasts and ion transporters).

A large number of studies have confirmed the important regulatory role of TGF-β1 in the process of amelogenesis. Normal regulation of TGF-β1 is beneficial to promote normal enamel secretion and mineralization, while abnormal regulation of TGF-β1 is associated with enamel hypoplasia. Enamel hypoplasia can further cause dental-related diseases and even affect patients’ quality of life. A deeper understanding of the regulatory roles of the TGF-β1 pathway in amelogenesis may help to reduce enamel destruction. Therefore, this review provides a comprehensive overview of the role of the TGF-β1 pathway in amelogenesis, summarizing the currently available scientific evidence. The process of amelogenesis and the biological characteristics of TGF-β1 are briefly described, and the regulatory effects of TGF-β1 on ameloblasts, enamel matrix synthesis, and enamel mineralization are elucidated (Figure 2).

More studies are needed to determine the molecular mechanisms involved in TGF-β1 regulation during enamel development. Dental enamel is known to show periodic growth patterns, such as the cross-striations and the Retzius lines (Smith, 2006). Additionally, ameloblasts oscillate between smooth-ended and ruffle-ended morphologies every 8 h during enamel maturation (Zheng et al., 2011). These results suggest that the process of enamel development is related to circadian rhythm. The important role of the circadian clock in enamel formation has been briefly elaborated.

In this review, a large number of literature have demonstrated that there is a close relationship between TGF-β1 and circadian clock genes in different tissues. Moreover, the canonical Wnt signaling pathway plays a central role in the circadian rhythm (Lecarpentier et al., 2019; Vallée et al., 2020; Vallee et al., 2022). Experiments confirmed that circadian clock genes inhibited ameloblast differentiation and matrix degradation through the Wnt/β-catenin signaling pathway, and mediated osteogenic differentiation and proliferation of mouse bone marrow mesenchymal stem cells through the Wnt/β-catenin signaling pathway (Zheng et al., 2022; Zou et al., 2022). In addition, TGF-β1 is closely related to the classical Wnt signaling pathway and can activate the Wnt signaling pathway to regulate cell differentiation and tissue development (Lecarpentier et al., 2019). Since the Wnt signaling pathway is a key bridge between circadian rhythm and cell function, and TGF-β1 is an important regulator of Wnt signaling, it can be inferred that there is a close relationship between TGF-β1 and circadian rhythm during amelogenesis.

During tooth development, similar to TGF-β1, circadian clock genes BMAL1, CLOCK, PER1, and PER2 were found to be expressed in the tooth germ (Zheng et al., 2011). In the process of enamel development, the important effects of TGF-β1 on ameloblast proliferation and differentiation, enamel matrix synthesis, and enamel matrix mineralization have been elucidated in this review. Numerous studies have shown that the circadian clock also has an important role in enamel development. The circadian clock can affect ameloblast differentiation, adhesion, matrix protein expression, and enamel ion transport (Athanassiou-Papaefthymiou et al., 2011; Lacruz et al., 2012a; Zheng et al., 2013; Wu et al., 2024). The overlapping regulatory roles of TGF-β1 and the circadian clock prompt us to consider the relationship between them in regulating amelogenesis.

This review illustrates the importance of TGF-β1 in the process of amelogenesis. In addition, TGF-β1/SMAD signaling has the potential to save enamel hypoplasia induced by fluoride and sulfur dioxide exposure in rats (Lv et al., 2023b). Further, TGF-β1 may regulate NBCe1 and may participate in the occurrence of dental fluorosis through the classic TGF-β1/SMAD pathway and the unconventional ERK and JNK pathways (Lv et al., 2023a). These results suggest that the TGF-β1/SMAD signaling pathway may be a potential target for the treatment of enamel hypoplasia and enamel fluorosis. Future studies could further explore the interaction of TGF-β1 with circadian rhythm and other signaling pathways, such as Wnt, and how to enhance therapeutic efficacy through multi-target combination therapy strategies.

## References

[B1] Abbarin N. San Miguel S. Holcroft J. Iwasaki K. Ganss B. (2015). The enamel protein amelotin is a promoter of hydroxyapatite mineralization. J. Bone Min. Res. 30 (5), 775–785. 10.1002/jbmr.2411 25407797

[B2] Abramyan J. Geetha-Loganathan P. Šulcová M. Buchtová M. (2021). Role of cell death in cellular processes during odontogenesis. Front. Cell Dev. Biol. 9, 671475. 10.3389/fcell.2021.671475 34222243 PMC8250436

[B3] Athanassiou-Papaefthymiou M. Kim D. Harbron L. Papagerakis S. Schnell S. Harada H. (2011). Molecular and circadian controls of ameloblasts. Eur. J. Oral Sci. 119 Suppl 1 (Suppl. 1), 35–40. 10.1111/j.1600-0722.2011.00918.x 22243224 PMC3516856

[B4] Aurrekoetxea M. Irastorza I. García-Gallastegui P. Jiménez-Rojo L. Nakamura T. Yamada Y. (2016). Wnt/β-Catenin regulates the activity of epiprofin/Sp6, SHH, FGF, and BMP to coordinate the stages of odontogenesis. Front. Cell Dev. Biol. 4, 25. 10.3389/fcell.2016.00025 27066482 PMC4811915

[B5] Balic A. Thesleff I. (2015). Tissue interactions regulating tooth development and renewal. Curr. Top. Dev. Biol. 115, 157–186. 10.1016/bs.ctdb.2015.07.006 26589925

[B6] Barcellos-Hoff M. H. Dix T. A. (1996). Redox-mediated activation of latent transforming growth factor-beta 1. Mol. Endocrinol. 10 (9), 1077–1083. 10.1210/mend.10.9.8885242 8885242

[B7] Bartlett J. D. Ryu O. H. Xue J. Simmer J. P. Margolis H. C. (1998). Enamelysin mRNA displays a developmentally defined pattern of expression and encodes a protein which degrades amelogenin. Connect. Tissue Res. 39 (1-3), 101–149. ; discussion 141-109. 10.3109/03008209809023916 11062992

[B8] Bartlett J. D. Simmer J. P. (2014). Kallikrein-related peptidase-4 (KLK4): role in enamel formation and revelations from ablated mice. Front. Physiol. 5, 240. 10.3389/fphys.2014.00240 25071586 PMC4082239

[B9] Bartlett J. D. Skobe Z. Nanci A. Smith C. E. (2011). Matrix metalloproteinase 20 promotes a smooth enamel surface, a strong dentino-enamel junction, and a decussating enamel rod pattern. Eur. J. Oral Sci. 119 Suppl 1 (Suppl. 1), 199–205. 10.1111/j.1600-0722.2011.00864.x 22243247 PMC3277084

[B10] Beynon A. L. Thome J. Coogan A. N. (2009). Age and time of day influences on the expression of transforming growth factor-beta and phosphorylated SMAD3 in the mouse suprachiasmatic and paraventricular nuclei. Neuroimmunomodulation 16 (6), 392–399. 10.1159/000228914 19609088

[B11] Bi R. Sun Y. Xiang L. Xu Z. Ye X. Tian Y. (2024). TGF-β1/Smad3 signaling is required to alleviate fluoride-induced enamel hypomineralization. Biol. Trace Elem. Res. 202 (2), 569–579. 10.1007/s12011-023-03688-y 37140770

[B12] Bromley K. M. Kiss A. S. Lokappa S. B. Lakshminarayanan R. Fan D. Ndao M. (2011). Dissecting amelogenin protein nanospheres: characterization of metastable oligomers. J. Biol. Chem. 286 (40), 34643–34653. 10.1074/jbc.M111.250928 21840988 PMC3186391

[B13] Caterina J. J. Skobe Z. Shi J. Ding Y. Simmer J. P. Birkedal-Hansen H. (2002). Enamelysin (matrix metalloproteinase 20)-deficient mice display an amelogenesis imperfecta phenotype. J. Biol. Chem. 277 (51), 49598–49604. 10.1074/jbc.M209100200 12393861

[B14] Chai Y. Zhao J. Mogharei A. Xu B. Bringas P. Jr. Shuler C. (1999). Inhibition of transforming growth factor-beta type II receptor signaling accelerates tooth formation in mouse first branchial arch explants. Mech. Dev. 86 (1-2), 63–74. 10.1016/s0925-4773(99)00112-4 10446266

[B15] Chen G. Deng C. Li Y. P. (2012). TGF-β and BMP signaling in osteoblast differentiation and bone formation. Int. J. Biol. Sci. 8 (2), 272–288. 10.7150/ijbs.2929 22298955 PMC3269610

[B16] Chen L. S. Couwenhoven R. I. Hsu D. Luo W. Snead M. L. (1992). Maintenance of amelogenin gene expression by transformed epithelial cells of mouse enamel organ. Arch. Oral Biol. 37 (10), 771–778. 10.1016/0003-9969(92)90110-t 1444889

[B17] Chen P. Kakan X. Wang S. Dong W. Jia A. Cai C. (2013). Deletion of clock gene Per2 exacerbates cholestatic liver injury and fibrosis in mice. Exp. Toxicol. Pathol. 65 (4), 427–432. 10.1016/j.etp.2011.12.007 22261359

[B18] Cho A. Haruyama N. Hall B. Danton M. J. Zhang L. Arany P. (2013). TGF-ß regulates enamel mineralization and maturation through KLK4 expression. PLoS One 8 (11), e82267. 10.1371/journal.pone.0082267 24278477 PMC3835418

[B19] Coin R. Haïkel Y. Ruch J. V. (1999). Effects of apatite, transforming growth factor beta-1, bone morphogenetic protein-2 and interleukin-7 on ameloblast differentiation *in vitro* . Eur. J. Oral Sci. 107 (6), 487–495. 10.1046/j.0909-8836.1999.eos107611.x 10625109

[B20] Danesi A. L. Athanasiadou D. Mansouri A. Phen A. Neshatian M. Holcroft J. (2021). Uniaxial hydroxyapatite growth on a self-assembled protein scaffold. Int. J. Mol. Sci. 22 (22), 12343. 10.3390/ijms222212343 34830225 PMC8620880

[B21] David C. J. Massagué J. (2018). Contextual determinants of TGFβ action in development, immunity and cancer. Nat. Rev. Mol. Cell Biol. 19 (7), 419–435. 10.1038/s41580-018-0007-0 29643418 PMC7457231

[B22] Deakins M. Volker J. F. (1941). Amount of organic matter in enamel from several types of human teeth. J. Dent. Res. 20 (2), 117–121. 10.1177/00220345410200020201

[B23] Derynck R. Zhang Y. E. (2003). Smad-dependent and Smad-independent pathways in TGF-β family signalling. Nature 425 (6958), 577–584. 10.1038/nature02006 14534577

[B24] Dong C. Gongora R. Sosulski M. L. Luo F. Sanchez C. G. (2016). Regulation of transforming growth factor-beta1 (TGF-β1)-induced pro-fibrotic activities by circadian clock gene BMAL1. Respir. Res. 17, 4. 10.1186/s12931-016-0320-0 26753996 PMC5477854

[B25] Dong J. Ruan W. Duan X. (2023). Molecular-based phenotype variations in amelogenesis imperfecta. Oral Dis. 29 (6), 2334–2365. 10.1111/odi.14599 37154292

[B26] Eckstein M. Lacruz R. S. (2018). CRAC channels in dental enamel cells. Cell Calcium 75, 14–20. 10.1016/j.ceca.2018.07.012 30114531 PMC6435299

[B27] Eckstein M. Vaeth M. Fornai C. Vinu M. Bromage T. G. Nurbaeva M. K. (2017). Store-operated Ca(2+) entry controls ameloblast cell function and enamel development. JCI Insight 2 (6), e91166. 10.1172/jci.insight.91166 28352661 PMC5358480

[B28] Franceschi R. T. Xiao G. (2003). Regulation of the osteoblast-specific transcription factor, Runx2: responsiveness to multiple signal transduction pathways. J. Cell Biochem. 88 (3), 446–454. 10.1002/jcb.10369 12532321

[B29] Fukae M. Tanabe T. Uchida T. Lee S. K. Ryu O. H. Murakami C. (1998). Enamelysin (matrix metalloproteinase-20): localization in the developing tooth and effects of pH and calcium on amelogenin hydrolysis. J. Dent. Res. 77 (8), 1580–1588. 10.1177/00220345980770080501 9719031

[B30] Fukumoto S. Kiba T. Hall B. Iehara N. Nakamura T. Longenecker G. (2004). Ameloblastin is a cell adhesion molecule required for maintaining the differentiation state of ameloblasts. J. Cell Biol. 167 (5), 973–983. 10.1083/jcb.200409077 15583034 PMC2172447

[B31] Fukumoto S. Yamada A. Nonaka K. Yamada Y. (2005). Essential roles of ameloblastin in maintaining ameloblast differentiation and enamel formation. Cells Tissues Organs 181 (3-4), 189–195. 10.1159/000091380 16612084

[B32] Gabe C. M. Bui A. T. Lukashova L. Verdelis K. Vasquez B. Beniash E. (2024). Role of amelogenin phosphorylation in regulating dental enamel formation. Matrix Biol. 131, 17–29. 10.1016/j.matbio.2024.05.004 38759902 PMC11363587

[B33] Gao Y. Li D. Han T. Sun Y. Zhang J. (2009). TGF-beta1 and TGFBR1 are expressed in ameloblasts and promote MMP20 expression. Anat. Rec. Hob. 292 (6), 885–890. 10.1002/ar.20901 19462458

[B34] Gao Y. Zhang L. Xiang L. Li B. Liu X. Wang Y. (2014). Transforming growth factor-β1 regulates expression of the matrix metalloproteinase 20 (Mmp20) gene through a mechanism involving the transcription factor, myocyte enhancer factor-2C, in ameloblast lineage cells. Eur. J. Oral Sci. 122 (2), 114–120. 10.1111/eos.12115 24495128

[B35] Gekakis N. Staknis D. Nguyen H. B. Davis F. C. Wilsbacher L. D. King D. P. (1998). Role of the CLOCK protein in the mammalian circadian mechanism. Science 280 (5369), 1564–1569. 10.1126/science.280.5369.1564 9616112

[B36] Gibson C. W. Yuan Z. A. Hall B. Longenecker G. Chen E. Thyagarajan T. (2001). Amelogenin-deficient mice display an amelogenesis imperfecta phenotype. J. Biol. Chem. 276 (34), 31871–31875. 10.1074/jbc.M104624200 11406633

[B37] Halder S. K. Beauchamp R. D. Datta P. K. (2005). Smad7 induces tumorigenicity by blocking TGF-beta-induced growth inhibition and apoptosis. Exp. Cell Res. 307 (1), 231–246. 10.1016/j.yexcr.2005.03.009 15922743

[B38] Haruyama N. Thyagarajan T. Skobe Z. Wright J. T. Septier D. Sreenath T. L. (2006). Overexpression of transforming growth factor-beta1 in teeth results in detachment of ameloblasts and enamel defects. Eur. J. Oral Sci. 114 Suppl 1, 30–379. ; discussion 39-41, 379. 10.1111/j.1600-0722.2006.00276.x 16674659

[B39] Hashem A. Kelly A. O'Connell B. O'Sullivan M. (2013). Impact of moderate and severe hypodontia and amelogenesis imperfecta on quality of life and self-esteem of adult patients. J. Dent. 41 (8), 689–694. 10.1016/j.jdent.2013.06.004 23778130

[B40] Hastings M. H. Reddy A. B. Maywood E. S. (2003). A clockwork web: circadian timing in brain and periphery, in health and disease. Nat. Rev. Neurosci. 4 (8), 649–661. 10.1038/nrn1177 12894240

[B41] He P. Zhang Y. Kim S. O. Radlanski R. J. Butcher K. Schneider R. A. (2010). Ameloblast differentiation in the human developing tooth: effects of extracellular matrices. Matrix Biol. 29 (5), 411–419. 10.1016/j.matbio.2010.03.001 20211728 PMC3296366

[B42] Hemeryck L. Hermans F. Chappell J. Kobayashi H. Lambrechts D. Lambrichts I. (2022). Organoids from human tooth showing epithelial stemness phenotype and differentiation potential. Cell Mol. Life Sci. 79 (3), 153. 10.1007/s00018-022-04183-8 35217915 PMC8881251

[B43] Howe K. L. Wang A. Hunter M. M. Stanton B. A. McKay D. M. (2004). TGFbeta down-regulation of the CFTR: a means to limit epithelial chloride secretion. Exp. Cell Res. 298 (2), 473–484. 10.1016/j.yexcr.2004.04.026 15265695

[B44] Hu C. C. Hart T. C. Dupont B. R. Chen J. J. Sun X. Qian Q. (2000). Cloning human enamelin cDNA, chromosomal localization, and analysis of expression during tooth development. J. Dent. Res. 79 (4), 912–919. 10.1177/00220345000790040501 10831092

[B45] Hu J. C. Sun X. Zhang C. Liu S. Bartlett J. D. Simmer J. P. (2002). Enamelysin and kallikrein-4 mRNA expression in developing mouse molars. Eur. J. Oral Sci. 110 (4), 307–315. 10.1034/j.1600-0722.2002.21301.x 12206593

[B46] Hu J. C. Zhang C. H. Yang Y. Kärrman-Mårdh C. Forsman-Semb K. Simmer J. P. (2001). Cloning and characterization of the mouse and human enamelin genes. J. Dent. Res. 80 (3), 898–902. 10.1177/00220345010800031001 11379892

[B47] Hu Y. Smith C. E. Cai Z. Donnelly L. A. Yang J. Hu J. C. (2016a). Enamel ribbons, surface nodules, and octacalcium phosphate in C57BL/6 *Amelx* ^-/-^ mice and *Amelx* ^+/-^ lyonization. Mol. Genet. Genomic Med. 4 (6), 641–661. 10.1002/mgg3.252 27896287 PMC5118209

[B48] Hu Y. Smith C. E. Richardson A. S. Bartlett J. D. Hu J. C. Simmer J. P. (2016b). MMP20, KLK4, and MMP20/KLK4 double null mice define roles for matrix proteases during dental enamel formation. Mol. Genet. Genomic Med. 4 (2), 178–196. 10.1002/mgg3.194 27066511 PMC4799876

[B49] Huang W. Zheng X. Yang M. Li R. Song Y. (2021). PER2-mediated ameloblast differentiation via PPARγ/AKT1/β-catenin axis. Int. J. Oral Sci. 13 (1), 16. 10.1038/s41368-021-00123-7 34011974 PMC8134554

[B50] Ikeda Y. Holcroft J. Ikeda E. Ganss B. (2022). Amelotin promotes mineralization and adhesion in collagen-based systems. Cell Mol. Bioeng. 15 (3), 245–254. 10.1007/s12195-022-00722-2 35611164 PMC9124263

[B51] Ikeda Y. Neshatian M. Holcroft J. Ganss B. (2018). The enamel protein ODAM promotes mineralization in a collagen matrix. Connect. Tissue Res. 59 (Suppl. 1), 62–66. 10.1080/03008207.2017.1408603 29745811

[B52] Ji M. Duan X. Han X. Sun J. Zhang D. (2021). Exogenous transforming growth factor-β1 prevents the inflow of fluoride to ameleoblasts through regulation of voltage-gated chloride channels 5 and 7. Exp. Ther. Med. 21 (6), 615. 10.3892/etm.2021.10047 33936272 PMC8082615

[B53] Ji M. Xiao L. Xu L. Huang S. Zhang D. (2018). How pH is regulated during amelogenesis in dental fluorosis. Exp. Ther. Med. 16 (5), 3759–3765. 10.3892/etm.2018.6728 30402142 PMC6201052

[B54] Kallenbach E. (1973). The fine structure of Tomes' process of rat incisor ameloblasts and its relationship to the elaboration of enamel. Tissue Cell 5 (3), 501–524. 10.1016/s0040-8166(73)80041-2 4744683

[B55] Kallenbach E. (1974). Fine structure of rat incisor ameloblasts in transition between enamel secretion and maturation stages. Tissue Cell 6 (1), 173–190. 10.1016/0040-8166(74)90030-5 4208837

[B56] Karamanos N. K. Theocharis A. D. Piperigkou Z. Manou D. Passi A. Skandalis S. S. (2021). A guide to the composition and functions of the extracellular matrix. Febs J. 288 (24), 6850–6912. 10.1111/febs.15776 33605520

[B57] Kawase T. Okuda K. Yoshie H. Burns D. M. (2002). Anti-TGF-beta antibody blocks enamel matrix derivative-induced upregulation of p21WAF1/cip1 and prevents its inhibition of human oral epithelial cell proliferation. J. Periodontal Res. 37 (4), 255–262. 10.1034/j.1600-0765.2002.01615.x 12200968

[B58] Kegulian N. C. Visakan G. Bapat R. A. Moradian-Oldak J. (2024). Ameloblastin and its multifunctionality in amelogenesis: a review. Matrix Biol. 131, 62–76. 10.1016/j.matbio.2024.05.007 38815936 PMC11218920

[B59] Kimchi A. Wang X. F. Weinberg R. A. Cheifetz S. Massagué J. (1988). Absence of TGF-beta receptors and growth inhibitory responses in retinoblastoma cells. Science 240 (4849), 196–199. 10.1126/science.2895499 2895499

[B60] Kingsley D. M. (1994). The TGF-beta superfamily: new members, new receptors, and new genetic tests of function in different organisms. Genes Dev. 8 (2), 133–146. 10.1101/gad.8.2.133 8299934

[B61] Kobayashi-Kinoshita S. Yamakoshi Y. Onuma K. Yamamoto R. Asada Y. (2016). TGF-β1 autocrine signalling and enamel matrix components. Sci. Rep. 6, 33644. 10.1038/srep33644 27633089 PMC5025654

[B62] Kubiczkova L. Sedlarikova L. Hajek R. Sevcikova S. (2012). TGF-β - an excellent servant but a bad master. J. Transl. Med. 10, 183. 10.1186/1479-5876-10-183 22943793 PMC3494542

[B63] Lacruz R. S. Hacia J. G. Bromage T. G. Boyde A. Lei Y. Xu Y. (2012a). The circadian clock modulates enamel development. J. Biol. Rhythms 27 (3), 237–245. 10.1177/0748730412442830 22653892 PMC3511783

[B64] Lacruz R. S. Nanci A. Kurtz I. Wright J. T. Paine M. L. (2010). Regulation of pH during amelogenesis. Calcif. Tissue Int. 86 (2), 91–103. 10.1007/s00223-009-9326-7 20016979 PMC2809306

[B65] Lacruz R. S. Smith C. E. Bringas P. Jr. Chen Y. B. Smith S. M. Snead M. L. (2012b). Identification of novel candidate genes involved in mineralization of dental enamel by genome-wide transcript profiling. J. Cell Physiol. 227 (5), 2264–2275. 10.1002/jcp.22965 21809343 PMC3243804

[B66] Lai C. F. Cheng S. L. (2002). Signal transductions induced by bone morphogenetic protein-2 and transforming growth factor-beta in normal human osteoblastic cells. J. Biol. Chem. 277 (18), 15514–15522. 10.1074/jbc.M200794200 11854297

[B67] Lecarpentier Y. Schussler O. Hébert J. L. Vallée A. (2019). Multiple targets of the canonical WNT/β-Catenin signaling in cancers. Front. Oncol. 9, 1248. 10.3389/fonc.2019.01248 31803621 PMC6876670

[B68] Lee D. J. Kim P. Kim H. Y. Park J. Lee S. J. An H. (2024). MAST4 regulates stem cell maintenance with DLX3 for epithelial development and amelogenesis. Exp. Mol. Med. 56 (7), 1606–1619. 10.1038/s12276-024-01264-5 38945953 PMC11297042

[B69] Lee H. K. Lee D. S. Ryoo H. M. Park J. T. Park S. J. Bae H. S. (2010). The odontogenic ameloblast-associated protein (ODAM) cooperates with RUNX2 and modulates enamel mineralization via regulation of MMP-20. J. Cell Biochem. 111 (3), 755–767. 10.1002/jcb.22766 20665536

[B70] Li S. Pan Y. (2017). Differential expression of transforming growth factor-beta1, connective tissue growth factor, phosphorylated-SMAD2/3 and phosphorylated-ERK1/2 during mouse tooth development. J. Mol. Histol. 48 (5-6), 347–355. 10.1007/s10735-017-9733-4 28825193

[B71] Li S. Pan Y. (2018). Immunolocalization of connective tissue growth factor, transforming growth factor-beta1 and phosphorylated-SMAD2/3 during the postnatal tooth development and formation of junctional epithelium. Ann. Anat. 216, 52–59. 10.1016/j.aanat.2017.10.005 29175126

[B72] Li Y. Lü X. Sun X. Bai S. Li S. Shi J. (2011). Odontoblast-like cell differentiation and dentin formation induced with TGF-β1. Arch. Oral Biol. 56 (11), 1221–1229. 10.1016/j.archoralbio.2011.05.002 21641578

[B73] Li Y. Zhou Y. Zhao C. Liu L. He Q. Shang K. (2024). The circadian clock gene, BMAL1, promotes radiosensitization in nasopharyngeal carcinoma by inhibiting the epithelial-to-mesenchymal transition via the TGF-β1/Smads/Snail1 axis. Oral Oncol. 152, 106798. 10.1016/j.oraloncology.2024.106798 38615583

[B74] Liang T. Hu Y. Smith C. E. Richardson A. S. Zhang H. Yang J. (2019). AMBN mutations causing hypoplastic amelogenesis imperfecta and Ambn knockout-NLS-lacZ knockin mice exhibiting failed amelogenesis and Ambn tissue-specificity. Mol. Genet. Genomic Med. 7 (9), e929. 10.1002/mgg3.929 31402633 PMC6732285

[B75] Lin C. Q. Bissell M. J. (1993). Multi-faceted regulation of cell differentiation by extracellular matrix. Faseb J. 7 (9), 737–743. 10.1096/fasebj.7.9.8330681 8330681

[B76] Liu X. Xu C. Tian Y. Sun Y. Zhang J. Bai J. (2019). RUNX2 contributes to TGF-β1-induced expression of Wdr72 in ameloblasts during enamel mineralization. Biomed. Pharmacother. 118, 109235. 10.1016/j.biopha.2019.109235 31336344

[B77] Lu T. Li M. Xu X. Xiong J. Huang C. Zhang X. (2018). Whole exome sequencing identifies an AMBN missense mutation causing severe autosomal-dominant amelogenesis imperfecta and dentin disorders. Int. J. Oral Sci. 10 (3), 26. 10.1038/s41368-018-0027-9 30174330 PMC6119682

[B78] Lu Y. Papagerakis P. Yamakoshi Y. Hu J. C. Bartlett J. D. Simmer J. P. (2008). Functions of KLK4 and MMP-20 in dental enamel formation. Biol. Chem. 389 (6), 695–700. 10.1515/bc.2008.080 18627287 PMC2688471

[B79] Lv Y. Wang W. Yao L. He J. Bai G. Lin C. (2023a). Sodium fluoride and sulfur dioxide derivatives induce TGF-β1-mediated NBCe1 downregulation causing acid-base disorder of LS8 cells. Biol. Trace Elem. Res. 201 (2), 828–842. 10.1007/s12011-022-03169-8 35304687

[B80] Lv Y. Wang Y. Yao J. He J. Lin C. Bai G. (2023b). The role of FGF9-mediated TGF-β1/Smad signaling in enamel hypoplasia induced by exposure to fluoride and SO(2) in rats. Ecotoxicol. Environ. Saf. 263, 115243. 10.1016/j.ecoenv.2023.115243 37454483

[B81] Lyons R. M. Keski-Oja J. Moses H. L. (1988). Proteolytic activation of latent transforming growth factor-beta from fibroblast-conditioned medium. J. Cell Biol. 106 (5), 1659–1665. 10.1083/jcb.106.5.1659 2967299 PMC2115066

[B82] Martinez-Avila O. Wu S. Kim S. J. Cheng Y. Khan F. Samudrala R. (2012). Self-assembly of filamentous amelogenin requires calcium and phosphate: from dimers via nanoribbons to fibrils. Biomacromolecules 13 (11), 3494–3502. 10.1021/bm300942c 22974364 PMC3496023

[B83] Massagué J. Sheppard D. (2023). TGF-β signaling in health and disease. Cell 186 (19), 4007–4037. 10.1016/j.cell.2023.07.036 37714133 PMC10772989

[B84] Meng X. M. Nikolic-Paterson D. J. Lan H. Y. (2016). TGF-β: the master regulator of fibrosis. Nat. Rev. Nephrol. 12 (6), 325–338. 10.1038/nrneph.2016.48 27108839

[B85] Miao X. Niibe K. Zhang M. Liu Z. Nattasit P. Ohori-Morita Y. (2021). Stage-specific role of amelx activation in stepwise ameloblast induction from mouse induced pluripotent stem cells. Int. J. Mol. Sci. 22 (13), 7195. 10.3390/ijms22137195 34281250 PMC8268366

[B86] Moffatt P. Smith C. E. St-Arnaud R. Nanci A. (2008). Characterization of Apin, a secreted protein highly expressed in tooth-associated epithelia. J. Cell Biochem. 103 (3), 941–956. 10.1002/jcb.21465 17647262

[B87] Moradian-Oldak J. George A. (2021). Biomineralization of enamel and dentin mediated by matrix proteins. J. Dent. Res. 100 (10), 1020–1029. 10.1177/00220345211018405 34151644 PMC8381691

[B88] Morkmued S. Hemmerle J. Mathieu E. Laugel-Haushalter V. Dabovic B. Rifkin D. B. (2017). Enamel and dental anomalies in latent-transforming growth factor beta-binding protein 3 mutant mice. Eur. J. Oral Sci. 125 (1), 8–17. 10.1111/eos.12328 28084688 PMC5260799

[B89] Moustakas A. Heldin C.-H. (2005). Non-Smad TGF-β signals. J. Cell Sci. 118 (16), 3573–3584. 10.1242/jcs.02554 16105881

[B90] Munger J. S. Harpel J. G. Giancotti F. G. Rifkin D. B. (1998). Interactions between growth factors and integrins: latent forms of transforming growth factor-beta are ligands for the integrin alphavbeta1. Mol. Biol. Cell 9 (9), 2627–2638. 10.1091/mbc.9.9.2627 9725916 PMC25536

[B91] Nagano T. Oida S. Suzuki S. Iwata T. Yamakoshi Y. Ogata Y. (2006). Porcine enamel protein fractions contain transforming growth factor-beta1. J. Periodontol. 77 (10), 1688–1694. 10.1902/jop.2006.050352 17032111

[B92] Nakata A. Kameda T. Nagai H. Ikegami K. Duan Y. Terada K. (2003). Establishment and characterization of a spontaneously immortalized mouse ameloblast-lineage cell line. Biochem. Biophys. Res. Commun. 308 (4), 834–839. 10.1016/s0006-291x(03)01467-0 12927794

[B93] Nakayama Y. Holcroft J. Ganss B. (2015). Enamel hypomineralization and structural defects in amelotin-deficient mice. J. Dent. Res. 94 (5), 697–705. 10.1177/0022034514566214 25715379

[B94] Nakayama Y. Tsuruya Y. Noda K. Yamazaki-Takai M. Iwai Y. Ganss B. (2019). Negative feedback by SNAI2 regulates TGFβ1-induced amelotin gene transcription in epithelial-mesenchymal transition. J. Cell Physiol. 234 (7), 11474–11489. 10.1002/jcp.27804 30488439

[B95] Nirvani M. Khuu C. Utheim T. P. Hollingen H. S. Amundsen S. F. Sand L. P. (2017). Circadian rhythms and gene expression during mouse molar tooth development. Acta Odontol. Scand. 75 (2), 144–153. 10.1080/00016357.2016.1271999 28030993

[B96] Niwa T. Yamakoshi Y. Yamazaki H. Karakida T. Chiba R. Hu J. C. (2018). The dynamics of TGF-β in dental pulp, odontoblasts and dentin. Sci. Rep. 8 (1), 4450. 10.1038/s41598-018-22823-7 29535349 PMC5849713

[B97] Ohta M. Greenberger J. S. Anklesaria P. Bassols A. Massagué J. (1987). Two forms of transforming growth factor-beta distinguished by multipotential haematopoietic progenitor cells. Nature 329 (6139), 539–541. 10.1038/329539a0 2889143

[B98] Oka S. Oka K. Xu X. Sasaki T. Bringas P. Jr. Chai Y. (2007). Cell autonomous requirement for TGF-beta signaling during odontoblast differentiation and dentin matrix formation. Mech. Dev. 124 (6), 409–415. 10.1016/j.mod.2007.02.003 17449229 PMC2704601

[B99] Okubo M. Chiba R. Karakida T. Yamazaki H. Yamamoto R. Kobayashi S. (2019). Potential function of TGF-β isoforms in maturation-stage ameloblasts. J. Oral Biosci. 61 (1), 43–54. 10.1016/j.job.2018.12.002 30929801

[B170] Papagerakis S. Zheng L. Schnell S. Sartor M. A. Somers E. Marder W. (2014). The circadian clock in oral health and diseases. J. Dent. Res. 93 (1), 27–35. 10.1177/0022034513505768 24065634 PMC3865791

[B100] Paine M. L. Snead M. L. (1997). Protein interactions during assembly of the enamel organic extracellular matrix. J. Bone Min. Res. 12 (2), 221–227. 10.1359/jbmr.1997.12.2.221 9041053

[B101] Palmer L. C. Newcomb C. J. Kaltz S. R. Spoerke E. D. Stupp S. I. (2008). Biomimetic systems for hydroxyapatite mineralization inspired by bone and enamel. Chem. Rev. 108 (11), 4754–4783. 10.1021/cr8004422 19006400 PMC2593885

[B102] Pandya M. Diekwisch T. G. H. (2021). Amelogenesis: transformation of a protein-mineral matrix into tooth enamel. J. Struct. Biol. 213 (4), 107809. 10.1016/j.jsb.2021.107809 34748943 PMC8665087

[B103] Pérez-Sala D. Rebollo A. (1999). Novel aspects of Ras proteins biology: regulation and implications. Cell Death Differ. 6 (8), 722–728. 10.1038/sj.cdd.4400557 10467345

[B104] Poché R. A. Sharma R. Garcia M. D. Wada A. M. Nolte M. J. Udan R. S. (2012). Transcription factor FoxO1 is essential for enamel biomineralization. PLoS One 7 (1), e30357. 10.1371/journal.pone.0030357 22291941 PMC3265481

[B105] Poulter J. A. Murillo G. Brookes S. J. Smith C. E. Parry D. A. Silva S. (2014). Deletion of ameloblastin exon 6 is associated with amelogenesis imperfecta. Hum. Mol. Genet. 23 (20), 5317–5324. 10.1093/hmg/ddu247 24858907 PMC4168819

[B106] Reith E. J. (1970). The stages of amelogenesis as observed in molar teeth of young rats. J. Ultrastruct. Res. 30 (1), 111–151. 10.1016/s0022-5320(70)90068-7 5411809

[B107] Reppert S. M. Weaver D. R. (2002). Coordination of circadian timing in mammals. Nature 418 (6901), 935–941. 10.1038/nature00965 12198538

[B108] Robinson C. Weatherell J. A. Hallsworth A. S. (1971). Variatoon in composition of dental enamel within thin ground tooth sections. Caries Res. 5 (1), 44–57. 10.1159/000259731 5278608

[B109] Russell W. E. Coffey R. J. Jr. Ouellette A. J. Moses H. L. (1988). Type beta transforming growth factor reversibly inhibits the early proliferative response to partial hepatectomy in the rat. Proc. Natl. Acad. Sci. U. S. A. 85 (14), 5126–5130. 10.1073/pnas.85.14.5126 3164865 PMC281701

[B110] Said R. Lobanova L. Papagerakis S. Papagerakis P. (2020). Calcium sets the clock in ameloblasts. Front. Physiol. 11, 920. 10.3389/fphys.2020.00920 32848861 PMC7411184

[B111] Sassá Benedete A. P. Sobral A. P. Lima D. M. Kamibeppu L. Soares F. A. Lourenço S. V. (2008). Expression of transforming growth factor-beta 1, -beta 2, and -beta 3 in human developing teeth: immunolocalization according to the odontogenesis phases. Pediatr. Dev. Pathol. 11 (3), 206–212. 10.2350/07-09-0333.1 18078367

[B112] Sato F. Sato H. Jin D. Bhawal U. K. Wu Y. Noshiro M. (2012). Smad3 and Snail show circadian expression in human gingival fibroblasts, human mesenchymal stem cell, and in mouse liver. Biochem. Biophys. Res. Commun. 419 (2), 441–446. 10.1016/j.bbrc.2012.02.076 22382019

[B113] Schmitz J. E. Teepe J. D. Hu Y. Smith C. E. Fajardo R. J. Chun Y. H. (2014). Estimating mineral changes in enamel formation by ashing/BSE and microCT. J. Dent. Res. 93 (3), 256–262. 10.1177/0022034513520548 24470541 PMC3929980

[B114] Schultz-Cherry S. Murphy-Ullrich J. E. (1993). Thrombospondin causes activation of latent transforming growth factor-beta secreted by endothelial cells by a novel mechanism. J. Cell Biol. 122 (4), 923–932. 10.1083/jcb.122.4.923 8349738 PMC2119591

[B115] Shaw W. J. Tarasevich B. J. Buchko G. W. Arachchige R. M. J. Burton S. D. (2020). Controls of nature: secondary, tertiary, and quaternary structure of the enamel protein amelogenin in solution and on hydroxyapatite. J. Struct. Biol. 212 (3), 107630. 10.1016/j.jsb.2020.107630 32979496 PMC7744360

[B116] Shi Y. Massagué J. (2003). Mechanisms of TGF-beta signaling from cell membrane to the nucleus. Cell 113 (6), 685–700. 10.1016/s0092-8674(03)00432-x 12809600

[B117] Simmer J. P. Hu J. C. Hu Y. Zhang S. Liang T. Wang S. K. (2021). A genetic model for the secretory stage of dental enamel formation. J. Struct. Biol. 213 (4), 107805. 10.1016/j.jsb.2021.107805 34715329 PMC8665125

[B118] Simmer J. P. Hu Y. Lertlam R. Yamakoshi Y. Hu J. C. (2009). Hypomaturation enamel defects in Klk4 knockout/LacZ knockin mice. J. Biol. Chem. 284 (28), 19110–19121. 10.1074/jbc.M109.013623 19578120 PMC2707199

[B119] Simmer J. P. Hu Y. Richardson A. S. Bartlett J. D. Hu J. C. (2011). Why does enamel in Klk4-null mice break above the dentino-enamel junction? Cells Tissues Organs 194 (2-4), 211–215. 10.1159/000324260 21546759 PMC3178080

[B120] Sire J. Y. Davit-Béal T. Delgado S. Gu X. (2007). The origin and evolution of enamel mineralization genes. Cells Tissues Organs 186 (1), 25–48. 10.1159/000102679 17627117

[B121] Smith C. E. (1998). Cellular and chemical events during enamel maturation. Crit. Rev. Oral Biol. Med. 9 (2), 128–161. 10.1177/10454411980090020101 9603233

[B122] Smith C. E. Issid M. Margolis H. C. Moreno E. C. (1996). Developmental changes in the pH of enamel fluid and its effects on matrix-resident proteinases. Adv. Dent. Res. 10 (2), 159–169. 10.1177/08959374960100020701 9206332

[B123] Smith C. E. Warshawsky H. (1975). Cellular renewal in the enamel organ and the odontoblast layer of the rat incisor as followed by radioautography using 3H-thymidine. Anat. Rec. 183 (4), 523–561. 10.1002/ar.1091830405 1200409

[B124] Smith C. E. Warshawsky H. (1977). Quantitative analysis of cell turnover in the enamel organ of the rat incisor. Evidence for ameloblast death immediately after enamel matrix secretion. Anat. Rec. 187 (1), 63–98. 10.1002/ar.1091870106 835843

[B125] Smith C. E. L. Kirkham J. Day P. F. Soldani F. McDerra E. J. Poulter J. A. (2017). A fourth KLK4 mutation is associated with enamel hypomineralisation and structural abnormalities. Front. Physiol. 8, 333. 10.3389/fphys.2017.00333 28611678 PMC5447068

[B126] Smith T. M. (2006). Experimental determination of the periodicity of incremental features in enamel. J. Anat. 208 (1), 99–113. 10.1111/j.1469-7580.2006.00499.x 16420383 PMC2100182

[B127] Snodgrass S. M. Cihil K. M. Cornuet P. K. Myerburg M. M. Swiatecka-Urban A. (2013). Tgf-β1 inhibits Cftr biogenesis and prevents functional rescue of ΔF508-Cftr in primary differentiated human bronchial epithelial cells. PLoS One 8 (5), e63167. 10.1371/journal.pone.0063167 23671668 PMC3650079

[B128] Song W. Wang Y. Chu Q. Qi C. Gao Y. Gao Y. (2018). Loss of transforming growth factor-β1 in epithelium cells affects enamel formation in mice. Arch. Oral Biol. 96, 146–154. 10.1016/j.archoralbio.2018.09.003 30243146

[B129] Stephanopoulos G. Garefalaki M. E. Lyroudia K. (2005). Genes and related proteins involved in amelogenesis imperfecta. J. Dent. Res. 84 (12), 1117–1126. 10.1177/154405910508401206 16304440

[B130] Sundell S. Valentin J. (1986). Hereditary aspects and classification of hereditary amelogenesis imperfecta. Community Dent. Oral Epidemiol. 14 (4), 211–216. 10.1111/j.1600-0528.1986.tb01537.x 3461907

[B131] Suzuki M. Shin M. Simmer J. P. Bartlett J. D. (2014). Fluoride affects enamel protein content via TGF-β1-mediated KLK4 inhibition. J. Dent. Res. 93 (10), 1022–1027. 10.1177/0022034514545629 25074495 PMC4212320

[B132] Takaguri A. Noro R. Shinohe S. Murayama R. Sakuraba M. Nomura R. (2025). Circadian clock gene BMAL1 is involved in transforming growth factor β1-induced fibrotic response in NRK-49F cells. Cell Biol. Int. 49 (4), 365–373. 10.1002/cbin.12273 39760204

[B133] Takahashi J. S. (2017). Transcriptional architecture of the mammalian circadian clock. Nat. Rev. Genet. 18 (3), 164–179. 10.1038/nrg.2016.150 27990019 PMC5501165

[B134] Takata T. D'Errico J. A. Atkins K. B. Berry J. E. Strayhorn C. Taichman R. S. (1998). Protein extracts of dentin affect proliferation and differentiation of osteoprogenitor cells *in vitro* . J. Periodontol. 69 (11), 1247–1255. 10.1902/jop.1998.69.11.1247 9848534

[B135] Tao J. Zhai Y. Park H. Han J. Dong J. Xie M. (2016). Circadian rhythm regulates development of enamel in mouse mandibular first molar. PLoS One 11 (8), e0159946. 10.1371/journal.pone.0159946 27494172 PMC4975438

[B136] Termine J. D. Belcourt A. B. Miyamoto M. S. Conn K. M. (1980). Properties of dissociatively extracted fetal tooth matrix proteins. II. Separation and purification of fetal bovine dentin phosphoprotein. J. Biol. Chem. 255 (20), 9769–9772. 10.1016/s0021-9258(18)43459-x 7430100

[B137] Tian Y. Mu H. Dong Z. Wang Y. Gao Y. Gao Y. (2022). The synergistic effects of TGF-β1 and RUNX2 on enamel mineralization through regulating ODAPH expression during the maturation stage. J. Mol. Histol. 53 (2), 483–492. 10.1007/s10735-022-10060-2 35165792

[B138] Tsuchiya M. Sharma R. Tye C. E. Sugiyama T. Bartlett J. D. (2009). Transforming growth factor-beta1 expression is up-regulated in maturation-stage enamel organ and may induce ameloblast apoptosis. Eur. J. Oral Sci. 117 (2), 105–112. 10.1111/j.1600-0722.2009.00612.x 19320718 PMC2711557

[B139] Tucker R. F. Shipley G. D. Moses H. L. Holley R. W. (1984). Growth inhibitor from BSC-1 cells closely related to platelet type beta transforming growth factor. Science 226 (4675), 705–707. 10.1126/science.6093254 6093254

[B140] Vaahtokari A. Vainio S. Thesleff I. (1991). Associations between transforming growth factor beta 1 RNA expression and epithelial-mesenchymal interactions during tooth morphogenesis. Development 113 (3), 985–994. 10.1242/dev.113.3.985 1726565

[B141] Vallee A. Lecarpentier Y. Vallée J. N. (2022). WNT/β-catenin pathway and circadian rhythms in obsessive-compulsive disorder. Neural Regen. Res. 17 (10), 2126–2130. 10.4103/1673-5374.332133 35259818 PMC9083179

[B142] Vallée A. Lecarpentier Y. Vallée R. Guillevin R. Vallée J. N. (2020). Circadian rhythms in exudative age-related macular degeneration: the key role of the canonical WNT/β-Catenin pathway. Int. J. Mol. Sci. 21 (3), 820. 10.3390/ijms21030820 32012797 PMC7037737

[B143] van der Kraan P. M. (2017). The changing role of TGFβ in healthy, ageing and osteoarthritic joints. Nat. Rev. Rheumatol. 13 (3), 155–163. 10.1038/nrrheum.2016.219 28148919

[B145] Wang J. Xiang H. Lu Y. Wu T. (2021). Role and clinical significance of TGF‑β1 and TGF‑βR1 in malignant tumors (Review). Int. J. Mol. Med. 47 (4), 55. (Review). 10.3892/ijmm.2021.4888 33604683 PMC7895515

[B146] Wang X. Chiba Y. Jia L. Yoshizaki K. Saito K. Yamada A. (2020). Expression patterns of claudin family members during tooth development and the role of claudin-10 (Cldn10) in cytodifferentiation of stratum intermedium. Front. Cell Dev. Biol. 8, 595593. 10.3389/fcell.2020.595593 33195274 PMC7642450

[B147] Wang Y. L. Lin H. C. Liang T. Lin J. C. Simmer J. P. Hu J. C. (2024). ENAM mutations can cause hypomaturation amelogenesis imperfecta. J. Dent. Res. 103 (6), 662–671. 10.1177/00220345241236695 38716742 PMC11122092

[B148] Wazen R. M. Moffatt P. Ponce K. J. Kuroda S. Nishio C. Nanci A. (2015). Inactivation of the Odontogenic ameloblast-associated gene affects the integrity of the junctional epithelium and gingival healing. Eur. Cell Mater 30, 187–199. 10.22203/ecm.v030a13 26412389

[B149] Weaver D. R. (1998). The suprachiasmatic nucleus: a 25-year retrospective. J. Biol. Rhythms 13 (2), 100–112. 10.1177/074873098128999952 9554572

[B150] Wu K. Li X. Bai Y. Heng B. C. Zhang X. Deng X. (2024). The circadian clock in enamel development. Int. J. Oral Sci. 16 (1), 56. 10.1038/s41368-024-00317-9 39242565 PMC11379899

[B151] Xie Z. Swain M. V. Hoffman M. J. (2009). Structural integrity of enamel: experimental and modeling. J. Dent. Res. 88 (6), 529–533. 10.1177/0022034509337130 19587157

[B152] Xing C. Zhai B. Zhang Y. Fang Y. Zhang M. Zhang C. (2024). Sleep deprivation reduced LPS-induced IgG2b production by up-regulating BMAL1 and CLOCK expression. Biochem. Biophys. Res. Commun. 691, 149326. 10.1016/j.bbrc.2023.149326 38035406

[B153] Yamada A. Yoshizaki K. Ishikawa M. Saito K. Chiba Y. Fukumoto E. (2021). Connexin 43-mediated gap junction communication regulates ameloblast differentiation via ERK1/2 phosphorylation. Front. Physiol. 12, 748574. 10.3389/fphys.2021.748574 34630166 PMC8500398

[B154] Yamakoshi Y. Richardson A. S. Nunez S. M. Yamakoshi F. Milkovich R. N. Hu J. C. (2011). Enamel proteins and proteases in Mmp20 and Klk4 null and double-null mice. Eur. J. Oral Sci. 119 Suppl 1 (Suppl. 1), 206–216. 10.1111/j.1600-0722.2011.00866.x 22243248 PMC3282035

[B155] Yamakoshi Y. Simmer J. P. Bartlett J. D. Karakida T. Oida S. (2013). MMP20 and KLK4 activation and inactivation interactions *in vitro* . Arch. Oral Biol. 58 (11), 1569–1577. 10.1016/j.archoralbio.2013.08.005 24112721 PMC3986046

[B156] Yang Y. Li Z. Chen G. Li J. Li H. Yu M. (2018). GSK3β regulates ameloblast differentiation via Wnt and TGF-β pathways. J. Cell Physiol. 233 (7), 5322–5333. 10.1002/jcp.26344 29215720

[B157] Yokozeki M. Afanador E. Nishi M. Kaneko K. Shimokawa H. Yokote K. (2003). Smad3 is required for enamel biomineralization. Biochem. Biophys. Res. Commun. 305 (3), 684–690. 10.1016/s0006-291x(03)00806-4 12763048

[B158] Yoshizaki K. Fukumoto S. Bikle D. D. Oda Y. (2020). Transcriptional regulation of dental epithelial cell fate. Int. J. Mol. Sci. 21 (23), 8952. 10.3390/ijms21238952 33255698 PMC7728066

[B159] Yu S. Zhang C. Zhu C. Quan J. Liu D. Wang X. (2022). A novel ENAM mutation causes hypoplastic amelogenesis imperfecta. Oral Dis. 28 (6), 1610–1619. 10.1111/odi.13877 33864320

[B160] Zarzynska J. M. (2014). Two faces of TGF-beta1 in breast cancer. Mediat. Inflamm. 2014, 141747. 10.1155/2014/141747 PMC4033515 24891760

[B161] Zhang X. L. Xi S. H. Cheng G. Y. Zhang Y. (2011). The effect of different concentrations of fluoride on the expression of Smad2/3 in ameloblast of rat incisor. Shanghai Kou Qiang Yi Xue 20 (3), 230–233. 21779727

[B162] Zheng J. Zhang L. Tan Z. Zhao Q. Wei X. Yang Y. (2022). Bmal1-and Per2-mediated regulation of the osteogenic differentiation and proliferation of mouse bone marrow mesenchymal stem cells by modulating the Wnt/β-catenin pathway. Mol. Biol. Rep. 49 (6), 4485–4501. 10.1007/s11033-022-07292-6 35386071

[B163] Zheng L. Papagerakis S. Schnell S. D. Hoogerwerf W. A. Papagerakis P. (2011). Expression of clock proteins in developing tooth. Gene Expr. Patterns 11 (3-4), 202–206. 10.1016/j.gep.2010.12.002 21156215 PMC3073654

[B164] Zheng L. Seon Y. J. Mourão M. A. Schnell S. Kim D. Harada H. (2013). Circadian rhythms regulate amelogenesis. Bone 55 (1), 158–165. 10.1016/j.bone.2013.02.011 23486183 PMC3650122

[B165] Zhong J. Shibata Y. (2022). The structural motifs of mineralized hard tissues from nano-to mesoscale: a future perspective for material science. Jpn. Dent. Sci. Rev. 58, 348–356. 10.1016/j.jdsr.2022.11.001 36404956 PMC9672955

[B166] Zhu H. Luo H. Shen Z. Hu X. Sun L. Zhu X. (2016). Transforming growth factor-β1 in carcinogenesis, progression, and therapy in cervical cancer. Tumour Biol. 37 (6), 7075–7083. 10.1007/s13277-016-5028-8 27010470

[B167] Zimmerman C. M. Padgett R. W. (2000). Transforming growth factor beta signaling mediators and modulators. Gene 249 (1-2), 17–30. 10.1016/s0378-1119(00)00162-1 10831835

[B168] Zoheiry M. M. Hasan S. A. El-Ahwany E. Nagy F. M. Taleb H. A. Nosseir M. (2015). Serum markers of epithelial mesenchymal transition as predictors of HCV-induced liver fibrosis, cirrhosis and hepatocellular carcinoma. Electron Physician 7 (8), 1626–1637. 10.19082/1626 26816590 PMC4725417

[B169] Zou T. Ma L. Gu L. Xi S. Zhang K. Guo X. (2022). Role of Wnt/β-catenin signaling pathway in ameloblast differentiation in relevance to dental fluorosis. Chem. Biol. Interact. 367, 110145. 10.1016/j.cbi.2022.110145 36063856

